# Choroidal thickness changes in children with chronic heart failure due to dilated cardiomyopathy

**DOI:** 10.1007/s10792-021-01774-5

**Published:** 2021-05-09

**Authors:** Klaudia Rakusiewicz, Krystyna Kanigowska, Wojciech Hautz, Lidia Ziółkowska

**Affiliations:** 1 Department of Ophthalmology, Children’s Memorial Health Institute, Warsaw, Poland; 2 Department of Cardiology, Children’s Memorial Health Institute, Warsaw, Poland

**Keywords:** Choroidal thickness, Optical coherence tomography, Dilated cardiomyopathy, Chronic heart failure

## Abstract

**Purpose:**

To evaluate choroidal thickness (CTh) in children with chronic heart failure (CHF) secondary to dilated cardiomyopathy (DCM) using spectral domain optical coherence tomography (SD-OCT) and to compare their values to those of healthy children.

**Methods:**

Sixty eyes of thirty children (mean age 9.9 ± 3.57 years) with chronic heart failure (left ventricular ejection fraction, LVEF ≤ 55%) due to DCM lasting for over 6 months were prospectively enrolled. The control group consisted of 30 age- (mean age 10.16 ± 3.42 years) and sex-matched healthy children. All participants underwent transthoracic echocardiography with LVEF measured using the Simpson method and had the blood serum level of *N*-terminal-pro-brain natriuretic peptide marker (NT-proBNP) determined. All children underwent SD-OCT and had subfoveal choroidal thickness (SFCTh) and CTh measured at 1500 µm (μm) nasally, temporally, superiorly and inferiorly from the fovea in both eyes by two investigators.

**Results:**

CTh at all locations was statistically significantly lower in children with DCM compared to the control group. Mean CTh in the group with CHF compared to the control group were (304.03 vs. 369.72 μm, *p* < 0.05) at the subfoveal location, (245.87 vs. 284 μm, *p* < 0.05) 1500 μm nasally from the fovea, (291.5 vs. 355.95 μm, *p* < 0.05) 1500 μm temporally from the fovea, (303.98 vs. 357.58 μm, *p* < 0.05) 1500 μm superiorly from the fovea and (290.92 vs. 344.96 μm, *p* < 0.05) 1500 μm inferiorly from the fovea. The average difference CTh between the study groups ranged from 38.13 to 65.69 μm at individual locations. In both groups, CTh was the thickest at subfoveal location (304.03 vs. 369.72 μm, *p* < 0.05) and the thinnest was 1500 μm nasally from the fovea (262.37 vs. 336.87 μm, *p* < 0.05). There was no correlation between CTh and age, gender, biometry and refractive error. No correlation was found between CTh and LVEF and NT-proBNP.

**Conclusion:**

Patients with CHF due to DCM had a thinner CTh at all measured locations. The results of our research indicate that CHF affects CTh and this parameter may be very helpful in monitoring the clinical course of the disease in children with DCM.

## Introduction

The choroid is the middle layer of the ocular wall, consisting mainly of a network of multiple calibre blood vessels [1]. The key choroidal function is to provide oxygen and nutrients to one-third of the external retina, including retinal pigment epithelium (RPE) and photoreceptors [2–4]. As the choroid is the only source of nutrients for the foveal avascular zone (FAZ), it ensures proper function of the most important of human senses [2, 4]. To ensure adequate blood supply to the retina, in particular to the photoreceptors, a high blood flow through the choroid is required [1]. The choroid is supplied by the three branches of the ocular artery: the short posterior ciliary arteries, the long posterior ciliary arteries and the anterior ciliary arteries [2]. The blood flow to the choroid is the highest of any tissue in the body per unit tissue weight [5] and is estimated to be 10 times higher than in the brain [1].

In the past, ocular ultrasound [6] and indocyanine-green angiography [7] were the only studies to enable choroidal assessment. However, none of them offers high accuracy and possibility to obtain cross-sectional scans of the choroid. The optical coherence tomography (OCT) has significantly improved the diagnostic possibilities for the choroid, and the development of the depth imaging technique—spectral domain optical coherence tomography (SD-OCT)—has enabled even more precise imaging alongside a qualitative and quantitative analysis [8]. CTh is an objective, measurable indicator of choroidal vasculature condition reflecting its circulatory performance [3, 9–11]. The OCT studies demonstrated that the mean subfoveal choroidal thickness (SFCTh) in healthy adults ranged from 272 μm to 448 μm [8, 12–15].

As the choroid mainly consists of blood vessel end branches, any disruption to the systemic circulation may adversely affect choroidal circulation [1, 2]. The choroid supplies retinal layers crucial for visual function, so blood vessel loss and choroidal thinning can lead to photoreceptor ischemia and, consequently, vision impairment or loss [16].

There is a plethora of studies to assess CTh in different conditions [8, 12–15, 17–20]. The changes in CTh have been confirmed in glaucoma [19], age-related macular degeneration [21], uveitis [18], retinitis pigmentosa [20] and myopia [17]. Similarly, there is evidence to support the effect of lupus nephritis [22], familial hypercholesterolaemia [23], diabetes mellitus [24], hypertension [25], cardiovascular diseases [3, 26] on CTh. However, little attention has been paid to date to assessing CTh changes in paediatric cardiovascular disorders leading to heart failure, such as cardiomyopathy.

Dilated cardiomyopathy (DCM) is associated with a reduced LVEF and an impaired oxygen supply disproportionately to tissue demand [27–29]. As a result, it is a significant cause of heart failure potentially leading to sudden cardiac death in children, with a very poor prognosis without a heart transplant [28, 29]. DCM is the most common paediatric cardiomyopathy type of multifactorial origin, with the incidence of about 0.58 cases per 100,000 children per year [28, 29]. Around 20 to 50% of children with DCM have a familial cardiomyopathy, where the condition affects a number of closer or more distant relatives [28–30]. However, the most common acquired cause of DCM in children is viral myocarditis which develops in predisposed individuals in response to a viral infection such as a common cold. Other causes of DCM include chemotherapy exposure and metabolic diseases. In many cases of idiopathic DCM, the causes remain undetermined [29]. The search for new testing methods and risk factors for adverse clinical outcomes in children with DCM is still ongoing. This prompted us to study the CTh as a non-invasive and readily available parameter monitoring the severity of haemodynamic changes in children with DCM. The aim of the study was to determine CTh values in children with CHF due to DCM using SD-OCT and compare them to those of healthy children.

## Materials and methods

This prospective study was conducted at the Department of Ophthalmology, Children's Memorial Health Institute in Warsaw between September 2019 and March 2020. It adhered to the tenets of the Declaration of Helsinki and was approved by the Bioethics Committee of the Children's Memorial Health Institute in Warsaw. All participants above 16 years of age and legal guardians of those below 16 years of age were provided explanations as to the nature and possible consequences of the study, and expressed their written, informed consent to participate in the study.

A total of 60 eyes of 30 children (16 male [M]/14 female [F], mean age 9.9 years ± 3.57; range 5–17) with chronic heart failure (CHF) due to DCM, treated in the Department of Cardiology at the Children's Memorial Health Institute, were enrolled. The study group inclusion criteria included confirmed CHF due to DCM lasting for over 6 months with LVEF ≤ 55%. The control group consisted of 60 eyes of 30 healthy children, without diagnosed heart failure or other systemic as well as ocular disease, matched for sex (16 M/14F) and age (mean age 10.16 ± 3.42; range 4–16). The exclusion criteria in both groups included ocular diseases, such as hereditary retinal dystrophy, glaucoma, uveitis, vitreoretinal diseases; previous ocular trauma, retinal laser photocoagulation, eye surgery, significant refractive error (spherical refractive error >  ± 3, cylindrical refractive error >  ± 3), significant systemic comorbidities and all other diseases with proven effect on CTh. Additionally, eyes with low quality scans were excluded.

**Fig. 1:**
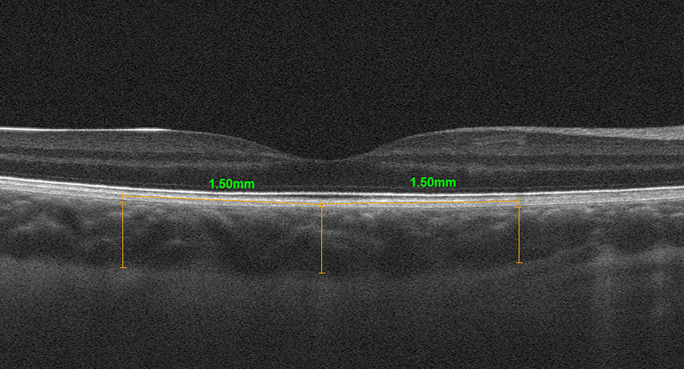
Manual measurement of choroidal thickness by the investigator using spectral domain optical coherence tomography

Clinical parameters collected in patients with DCM included serum level of N-terminal-pro-brain natriuretic peptide (NT-proBNP) and LVEF measured using the Simpson method during the two-dimensional transthoracic echocardiography. Each patient underwent a full ophthalmic assessment, including best-corrected visual acuity (BCVA) assessed with Snellen’s chart, anterior segment slit lamp biomicroscopy, fundus examination, ocular axial length measurement and cycloplegic (1% Tropicamide) refraction testing. The spectral domain OCT (SD-OCT) was performed in all participants using commercially available RTVue XR Avanti OCT system (Optovue, Fremont, California, United States). A crossline scan was performed to obtain high-quality images of retina and choroid. The CTh was measured manually, using a calliper in SD-OCT software. The CTh was defined as the distance between the hyperreflective line corresponding to the outer RPE border and the hyperreflective line corresponding to the inner scleral border. This parameter was measured by two independent investigators (KR and KK), and the mean of the two results was included in the analyses. The measurements were obtained in the subfoveal region, which was defined as the lowest point shown on retinal tomograms, and at 1500 μm superiorly, inferiorly, nasally and temporally from the fovea (Fig. 1). Since some studies point to diurnal variation in CTh, all measurements were taken at the same time of day in all children [31, 32]. Usui et al. [31] noted the mean reduction in CTh of 33.0 ± 14.3 μm between 9 am and 6 pm. Likewise, Tan et al. [32] observed a similar trend reporting the mean reduction in CTh of 33.7 ± 21.5 μm between 9 am and 5 pm, confirmed with two tests on separate visits. The reason for this diurnal CTh fluctuation remains unknown to date [31, 32]. In order to control for the potential effect of diurnal variation in CTh on the measurements obtained in our study, the subsequent SD-OCT scans were performed in a given child at the same time of the day, with appointments scheduled overall in all participants between 11 am and 3 pm. The data for both eyes were analysed. Eyes with poor quality, defocused scans or those where scleral margin was not visible were excluded.

## Statistical analysis

The presented variables were expressed as means, standard deviations, 95% confidence intervals and ranges. The two-way Mann–Whitney test for two independent samples was used to determine the presence of statistical differences between the study group and the controls. It is a nonparametric alternative to Student’s t test, which could not be used in the analysis due to the failure to meet the assumptions about the normal distribution of tested samples. Linear relationships between selected quantitative variables were calculated using the Pearson product-moment correlation coefficient. The level *p* < 0.05 was considered statistically significant for all calculated comparisons. All statistical analyses were performed using R 3.5.1 (R Core Team 2018).

## Results

**Fig. 2:**
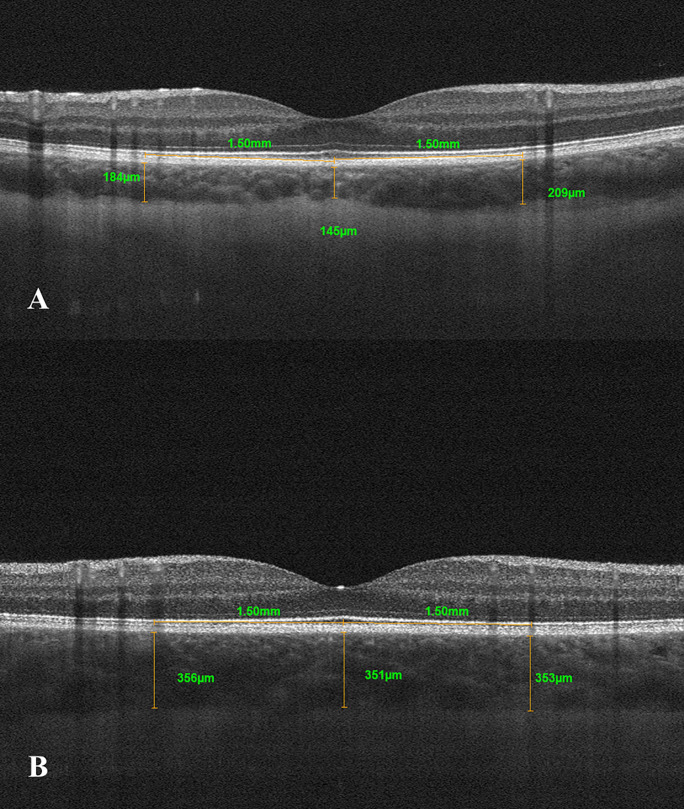
Manual measurement of choroidal thickness by the investigator using the spectral domain of coherent optical tomography (**a**) in a patient with dilated cardiomyopathy (**b**) in a control group

**Fig. 3:**
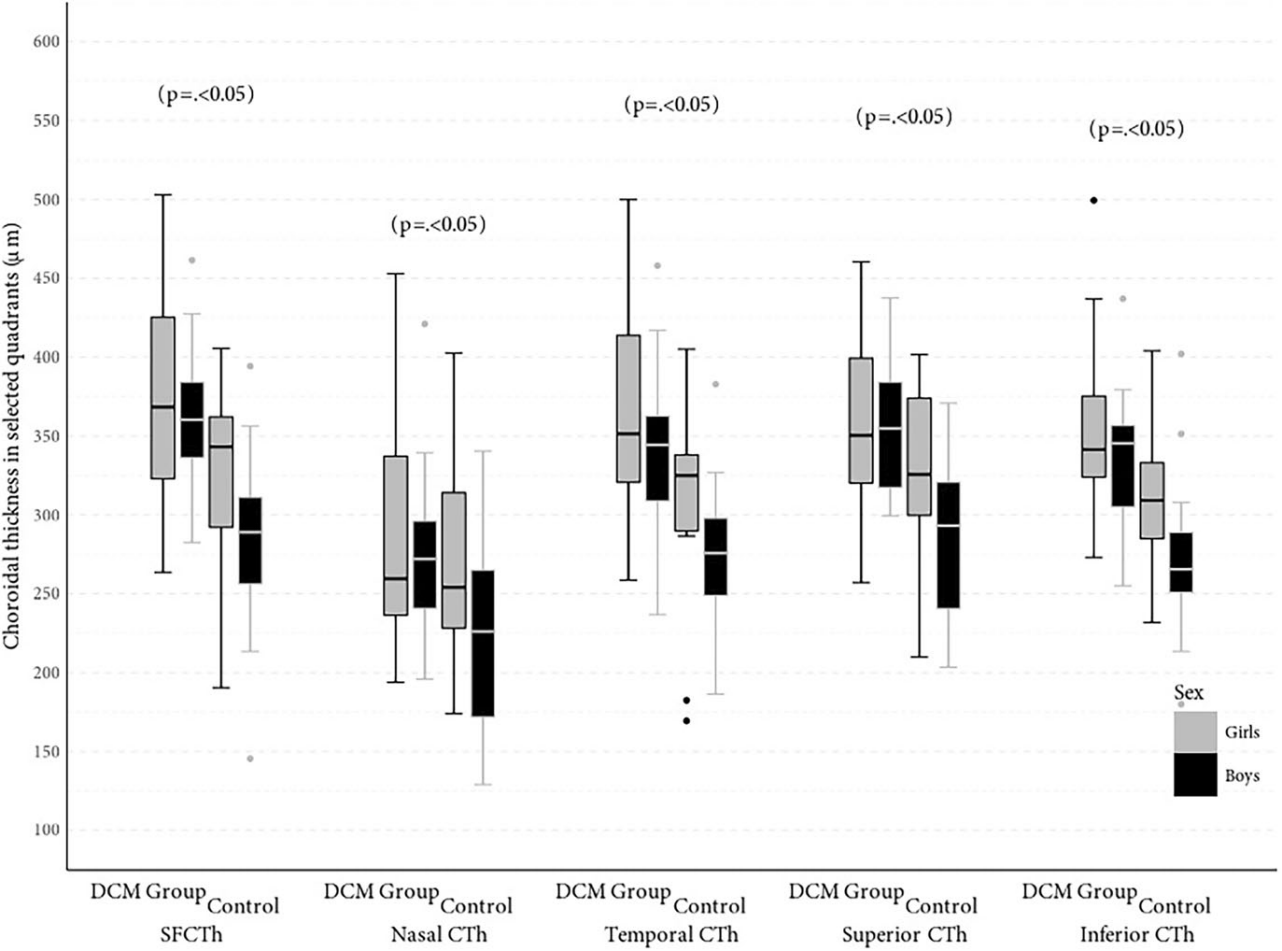
Central tendency and dispersion for choroidal thickness (μm) at selected anatomical locations in the study sample by DCM status and sex. The box is drawn from the first quartile to the third obtained data values, in the middle a thick horizontal line indicates a median. The whiskers are used to determine the minimum and maximum value: the lowest data point excluding any outliers is the minimum value, and the highest data point excluding any outliers is the maximum value

**Fig. 4:**
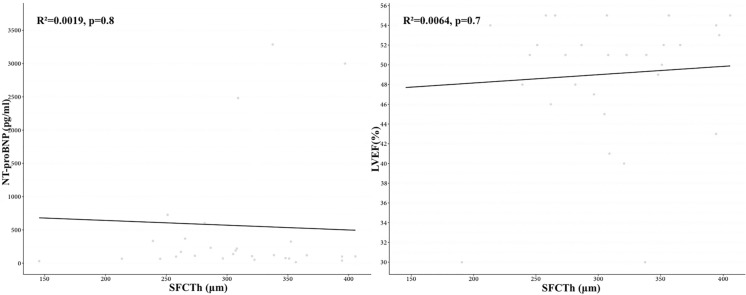
Graph of correlation between subfoveal choroidal thickness (SFCTh) and N-terminal (NT)-pro hormone BNP (NT-proBNP) and left ventricular ejection fraction (LVEF)

Data were collected for 60 eyes of 30 patients (16 M /14F) with CHF due to DCM and 60 eyes of 30 patients (16 M/14F) in the control group. The mean age was 9.9 ± 3.57 years (range 5–17 years) for patients with DCM and 10.16 ± 3.42 years (range 4–16 years) for controls (*p* = 0.68). Detailed characteristics of the study and control groups are presented in Table 1. Demographic data including age and gender were comparable in both groups. The mean value of LVEF in the DCM group was 49.03% (range 30–55%), and the mean value of NT-pro-BNP was 568.1 pg/mL (range 15–3723 pg/mL). All enrolled participants had full visual acuity (20/20) as assessed using the Snellen chart. The CTh in all locations included in the analysis was statistically significantly thinner in patients with DCM compared to the control group, and the results are presented in Table 2 (Fig. 2). Mean CTh in the group with DCM compared to the control group were (304.03 vs. 369.72 μm, *p* < 0.05) at the subfoveal location, (245.87 vs. 284 μm, *p* < 0.05) 1500 μm nasal to the fovea, (291.5 vs. 355.95 μm, *p* < 0.05) 1500 μm temporal to the fovea, (303.98 vs. 357.58 μm, *p* < 0.05) 1500 μm superior to the fovea, (290.92 vs. 344.96 μm, *p* < 0.05) 1500 μm inferior to the fovea (Fig. 3). The average difference between the groups ranged from 38.13 to 65.69 μm at individual locations. In both groups, CTh was the thickest at subfoveal location (304.03 vs. 369.72 μm, *p* < 0.05) and the thinnest was 1500 μm nasally from the fovea (245.87 vs. 284 μm, *p* < 0.05). There was no correlation between CTh and ophthalmic values such as biometry, refractive error, and age of subjects. It was noted that the choroid was thicker in the girls compared to the boys. The mean of SFCTh in boys was 322.92 ± 65.83 μm and in girls 355.68 ± 65.53 μm (*p* < 0.05). The CTh in DCM patients did not correlate with LVEF or NT-proBNP values (Fig. 4).

**Table 1:** Demographic and clinical features in dilated cardiomyopathy patients and control subjects

Variable	Study group	M	SD	95% CI	Range	*p*
NT-proBNP (pg/ml)	DCM group	568.10	1045.27	177.79–958.41	15–3723	–
LVEF (%)	DCM group	49.03	6.63	46.56–51.51	30–55	–
Age (years)	DCM group	9.9	3.57	8.57–11.23	5–17	0.68
	Control	10.16	3.42	9.03–11.28	4–16	
Biometry (mm)	DCM group	22.17	0.88	21.85–22.5	20.615–24.08	0.09
	Control	22.49	0.63	22.29–22.7	21.1–23.795	
Spherical refractive error	DCM group	0.75	1.14	0.32–1.17	− 2.00–3.00	0.45
	Control	0.56	1.18	0.17–0.94	− 2.25–2.75	
Cylindrical refractive error	DCM group	0.17	0.29	0.06–0.28	0.00–1.25	0.1
	Control	0.26	0.35	0.15–0.38	0.00–1.00	

*M* mean, *SD* standard deviation, *CI* confidence interval, *LVEF* left ventricular ejection fraction, *NT-proBNP* natriuretic peptide type B, *DCM group* group with dilated cardiomyopathy

**Table 2:** Choroidal thickness at individual locations in dilated cardiomyopathy patients and control subjects

Variable	Study Group	M	SD	95% CI	Range	*p*
SFCTh	DCM group	304.03	63.03	280.5–327.57	145.5–405.5	< 0.05
	Control group	369.72	55.79	351.39–388.06	263.5–503	
Nasal CTh	DCM group	245.87	68.28	220.37–271.36	129–402.5	< 0.05
	Control group	284	63.2	263.23–304.77	194–453	
Temporal CTh	DCM group	291.5	57.12	270.17–312.83	169.5–405	< 0.05
	Control group	355.95	61.77	335.64–376.25	237–500	
Superior CTh	DCM group	303.98	58.38	282.18–325.78	203.5–401.5	< 0.05
	Control group	357.58	50.87	340.86–374.3	257–460.5	
Inferior CTh	DCM group	290.92	52.59	271.28–310.55	180–404	< 0.05
	Control group	344.96	48.48	329.03–360.89	255–499.5	

*M* mean, *SD* standard deviation, *CI* confidence interval, *SFCTh* subfoveal choroidal thickness, *CTh* choroidal thickness

## Discussion

Amid the plethora of research assessing CTh in systemic diseases, no study has been conducted in patients with CHF due to DCM. To the best of our knowledge, ours is the first study to compare CTh between children with DCM and healthy controls.

DCM is the most common myocardial disease in children and the most common cause of heart failure in the paediatric population [27, 28]. Morbidity and mortality rates are high in children with DCM. Progressive heart failure in DCM is the most common indication for a heart transplant [29].

Although the available diagnostic armamentarium offers a number of accurate and reliable tests, there is still unfailing interest in novel methods to diagnose, monitor progression and guide treatment of systemic diseases in children. In paediatric care, the tests need to be non-invasive, quick and fairly easy to perform. The ability to easily and quickly image the choroid with SD-OCT offers new possibilities for quantitative assessment of choroidal thickness so as to determine choroidal circulatory status. The CTh measurement is highly reproducible between various investigators and devices [33].

Anatomically, the choroid consists of several vascular layers [1]. All choroidal vessels are terminal branches, which do not form anastomoses [2]. Therefore, any vascular dysfunction leads to their occlusion, due to the lack of collateral circulation [2]. The main underlying mechanism in DCM is left ventricular systolic dysfunction which plays a role in disease progression leading to exacerbation of heart failure symptoms [34]. As the left ventricle gets dilated, its walls become thinner [27]. Consequently, this makes the myocardium incapable of pumping blood into the systemic circulation and thereby to individual organs according to their physiological demand [34]. The LVEF and systemic arterial pressure drop, which causes hypoxia due to inadequate oxygen supply to peripheral organs, including the choroid [34]. As a result, the vascular network may lose its density, which manifests as choroidal thinning shown in our study.

There are published studies, which indicate the effect of circulatory conditions leading to heart failure (HF) on CTh. Altinkaynak et al. [10] measured CTh in 56 adult patients with heart failure. They noted a reduction in the mean subfoveal choroidal thickness (SFCTh) (181.2 ± 80.23 μm) in patients with HF compared to healthy controls (283.6 ± 52.4 μm, *p* = 0.000). There was a correlation between SFCTh and LVEF. Ahmad et al. [3] in their study in 34 patients with coronary artery disease (CAD) documented by the history of at least 50% obstruction in at least one coronary artery on cardiac catheterization, positive stress test, ST elevation myocardial infarction (STEMI), or revascularization, observed reduced SFCTh (252 vs. 303 μm, *p* = 0.002) and reduced CTh in all other measurement points (superior, inferior, nasal and temporal to fovea) in CAD patients as compared to controls. Based on the analysis of 158 patients with coronary heart disease (CHD), Wang et al. [35] reported a trend for decreasing vessel density and flow area in CHD patients compared to healthy subjects. There was a significant negative association between the severity of coronary artery stenosis and vessel density, including the choroid capillary fovea and choroid capillary flow. However, Doğan et al. [36] and Kanar et al. [37] found a thinner choroid in patients with coronary slow-flow phenomenon compared to healthy volunteers. Moreover, Schuster et al. [26] assessed the correlation between CTh and cardiovascular risk factors. Patients with reduced LVEF, higher systolic pressure and pulse pressure tended to have a thinner choroid. The results of our study demonstrating a reduced choroidal thickness in patients with heart failure support the above findings. On the other hand, there are studies, where no difference in SFCTh was demonstrated between patients with cardiomyopathy and healthy controls, for example the one by Bayramoğlu et al. [38], carried out in 33 patients with diastolic dysfunction secondary to hypertrophic cardiomyopathy or the one by Alur et al. [39] carried out in adults with DCM with LVEF below 40%.

A number of studies support the effect of age, sex, biometry and refractive errors on CTh [15, 40, 44] with age indicated as the strongest CTh modifier [12, 15, 45]. The choroid is usually significantly thicker in children than in adults [12, 40, 41]. The mean SFCTh in healthy adults ranges between 272 μm and 448 μm [8, 12–14] as compared to 302–388.2 μm [40, 41, 46–48] in children. It tends to get thinner from around the age of 30, decreasing on average by 16–20 μm every 10 years [42]. As pointed out by Barteselli et al. [43], choroidal volume also decreases by 0.54 mm^3^ (7.32%) per decade. Formerly, Sarks [49] and Ramrattan et al. [50] drew comparable conclusion based on histological studies. In our study, the correlation between CTh and age could not be demonstrated as our group was fairly young with a narrow age range, which would suggest the previous finding that there is no age-related reduction in choroidal thickness in the first or second decade of life [42].

Most studies show a higher CTh in men than in women [43–45]. Those sex-based differences may be due to a higher basal sympathetic tone and hormonal changes in women [51], however, as reported by Park et al. [40], the effect of hormones on CTh in children may not be significant. Other authors have not reported significant changes in the CTh according to sex [41, 46]. In our study, we found a statistically significant difference in the CTh between boys and girls. Girls had significantly thicker membranes compared to boys, which is not in line with the analyses presented above. On the contrary, Mapelli et al. [52] found that the choroidal volume is higher in girls than in boys. The significance of this result remains uncertain, and the exact mechanism underlying this finding is not clear. Our study may not be strong enough to detect the actual intersexual difference in choroidal thickness, so further studies involving more eyes are needed to confirm this relationship.

More recent studies have identified the link between CTh and the ocular axial length [15, 17, 41, 44]. He et al. [41] quantified the choroidal thickness reduction as 23 μm per each 1 mm of ocular length, whereas Li et al. [44] quantified it as 58.2 μm per each 1 mm of ocular length. The ocular axial length is strongly linked to the refractive error—myopic eyes are longer [53]. The finding of thinner choroid in myopic eyes as compared to emmetropic eyes is not surprising, then [15, 17, 45]. According to Ikuno et al. [54], the choroid in myopic eyes can be about three times thinner than in emmetropic eyes. Other authors observed CTh thinning in myopic eyes by 8.7 to 9.3 μm per every dioptre [15, 17, 45]. There are studies, finally, which do not report a correlation between CTh and ocular biometry or refractive errors. The results of our study are consistent with the above and did not reveal any correlation between CTh and either the ocular axial length or the refractive error. However, children with a high refractive error (defined as values above + 3.0 D or  − 3.0D) were excluded from our study, whereby subjects with very short and very long eyes were secondarily excluded. Thus, this may explain the lack of correlation.

In our study, we observed variable CTh, depending on topographic location in both enrolled groups. The choroid was the thickest in the subfoveal region (304.03 vs. 369.72 μm). Also, the CTh values measured at 1500 μm nasally from the fovea (245.87 vs. 284 μm) were lower than those measured at 1500 μm temporally from the fovea (291.5 vs. 355.95 μm). These results are in line with analyses of other authors [8, 46]. The differences in CTh between particular locations can be explained by the anatomy of the eye, as the optic disc is the site where the optic nerve exits the eye and ciliary arteries pierce the sclera to supply the choroid. As a result, the inferior peripapillary area has a thicker retina and a thinner choroid [15, 46]. On the contrary, the other authors did not observe a similar topographic distribution [17, 41].

Regardless of the above-mentioned factors, in our study, children with CHF had significantly thinner CTh as compared to healthy control. It may suggest that the reduced LVEF and stroke volume cause an impairment in choroidal circulation which, in turns, leads to choroidal thinning. Choroidal function, on the other hand, directly translates into retinal function and potential retinal pathologies. Therefore, a reduced CTh secondary to dysfunctional choroidal circulation may cause vision impairment or such posterior segment conditions as age-related macular degeneration and central serous retinopathy [2].

## Limitations

There are, however, some limitations to our study. First of them is the small study sample. Furthermore, the CTh measurements were performed manually, due to the lack of dedicated software enabling automated measurements. CTh readings were taken at the three selected locations, while more measurement sites were used in other published studies. Accurate, quantitative OCT-based analysis of CTh as the parameter reflecting the health of the choroid has been studied since the launch of the first OCT devices. Further research in a larger sample is needed, which will enable assessing the repeatability, sensitivity and specificity of the method so that OCT choroidal thickness measurement can be used in future to evaluate DCM severity and to facilitate clinical decision making.

## Summary

We evaluated CTh in a group of children with CHF due to DCM compared to healthy controls. There is a significant negative relationship between choroidal thickness and the presence of CHF due to DCM. This study provides important insight into the effect of cardiovascular disorders on choroidal vasculature. Reduced choroidal thickness may be an indicator of circulatory abnormalities in the choroid. Our study indicates that CTh measurement as a repetitive, non-invasive method may be an objective marker for monitoring disease progression and treatment in children with DCM. Further randomized studies are needed to fully determine the utility of CTh in children with DCM.
